# An Oncolytic Vaccinia Virus Expressing Aphrocallistes Vastus Lectin Modulates Hepatocellular Carcinoma Metabolism via ACSS2/TFEB-Mediated Autophagy and Lipid Accumulation

**DOI:** 10.3390/md23080297

**Published:** 2025-07-24

**Authors:** Qiang Wang, Simeng Zhou, Yin Wang, Yajun Gao, Yanrong Zhou, Ting Ye, Gongchu Li, Kan Chen

**Affiliations:** 1 College of Life Sciences and Medicine, Zhejiang Sci-Tech University, Hangzhou 310018, China; 15605243901@163.com (Q.W.); 18979357466@163.com (S.Z.); wangyin2002@outlook.com (Y.W.); zhouyanrong@zstu.edu.cn (Y.Z.); yeting@zstu.edu.cn (T.Y.); 2 Hangzhou Gongchu Biotechnology Co., Ltd., Hangzhou 310018, China; gaoyajun_2000@163.com

**Keywords:** Aphrocallistes vastus lectin, oncolytic vaccinia virus, autophagy, ACSS2

## Abstract

Hepatocellular carcinoma (HCC) remains a therapeutic challenge due to metabolic plasticity and drug resistance. Oncolytic viruses (OVs), such as thymidine kinase-deleted vaccinia virus (oncoVV), selectively lyse tumors while stimulating antitumor immunity, however, their metabolic interplay with cancer cells is poorly understood. Here, we engineered an oncoVV-expressing Aphrocallistes vastus lectin (oncoVV-AVL) and uncovered its unique ability to exploit the ACSS2/TFEB axis, driving metabolic competition in HCC. In vitro, oncoVV-AVL triggered cell autophagy and lipid accumulation (3.4–5.7-fold upregulation of FASN and ACC1) while suppressing glucose uptake (41–63% higher extracellular glucose and 33–34% reduced lactate). Mechanistically, oncoVV-AVL upregulated acetyl-CoA synthetase 2 (ACSS2), promoting its nuclear translocation and interaction with transcription factor EB (TFEB) to concurrently activate lipogenesis and autophagic flux. The pharmacological inhibition of ACSS2 abolished these effects, confirming its central role. In vivo, oncoVV-AVL suppressed tumor growth while inducing lipid deposition (2-fold triglyceride increase), systemic hypoglycemia (42% glucose reduction), and autophagy activation (elevated LC3B-II/I ratios). This study establishes ACSS2 as a metabolic checkpoint in OV therapy, providing a rationale for combining oncolytic virotherapy with metabolic modulators in HCC.

## 1. Introduction

Hepatocellular carcinoma (HCC), representing 75–85% of primary liver cancers, faces therapeutic challenges due to tumor heterogeneity and drug resistance [1]. Metabolic reprogramming, characterized by hyperactive glycolysis and persistent fatty acid synthesis, represents a hallmark of HCC and a promising therapeutic target [2,3].

Oncolytic viruses (OVs), particularly thymidine kinase (TK)-deficient variants, have emerged as a dual-pronged strategy combining tumor-specific lysis and immune activation. In the coming years, compounds derived from marine organisms may be an inspiring tool to develop new anticancer agents [4]. Aphrocallistes vastus lectin (AVL) is a marine sponge-derived C-type lectin [5]. Our previous studies demonstrated that oncolytic vaccinia virus carrying AVL (oncoVV-AVL) exerts a superior antitumor efficacy in multiple tumor models [6,7,8]. Mechanistically, oncoVV-AVL suppresses nuclear factor erythroid 2-related factor 2 (Nrf2) to elevate reactive oxygen species (ROS) levels, triggering oxidative stress and autophagy-associated cell death [7,8]. Previous studies demonstrated that viral replication relies on host lipid metabolism [9,10,11], suggesting a potential interplay between HCC’s inherent lipogenic dysregulation and oncoVV-AVL’s therapeutic mechanism—an underexplored dimension addressed herein.

Autophagy is an intracellular lysosome-mediated degradation process that plays a crucial role in maintaining cellular homeostasis through the mammalian target of rapamycin (mTOR)- and AMP-activated protein kinase (AMPK)-dependent regulation [12,13]. Under normal conditions, basal autophagy activity remains low; however, this activity is markedly enhanced under starvation or stress conditions. Recent studies verified that nutrient deprivation triggers the AMPK-mediated phosphorylation of acetyl-CoA synthetase 2 (ACSS2), promoting its nuclear translocation. Nuclear ACSS2 cooperates with transcription factor EB (TFEB) to concurrently activate autophagy–lysosomal genes and lipogenic programs via histone acetylation. This crosstalk positions fatty acid metabolism as a potential link in autophagy regulation, which has not been elucidated in the treatment of oncolytic vaccinia virus [14,15].

Here, we demonstrate that oncoVV-AVL triggers metabolic competition in HCC by regulating the ACSS2/TFEB axis, simultaneously disrupting glucose utilization and coupling lipogenesis to autophagic flux. By expanding the mechanistic paradigm of OVs beyond cytolysis and immune stimulation, our findings provide a rationale for combining oncolytic virotherapy with metabolic inhibitors (e.g., AMPK agonists) in tumor treatment.

## 2. Results

### 2.1. OncoVV-AVL Induces Lipid Accumulation in HCC Cells

**Figure 1:**
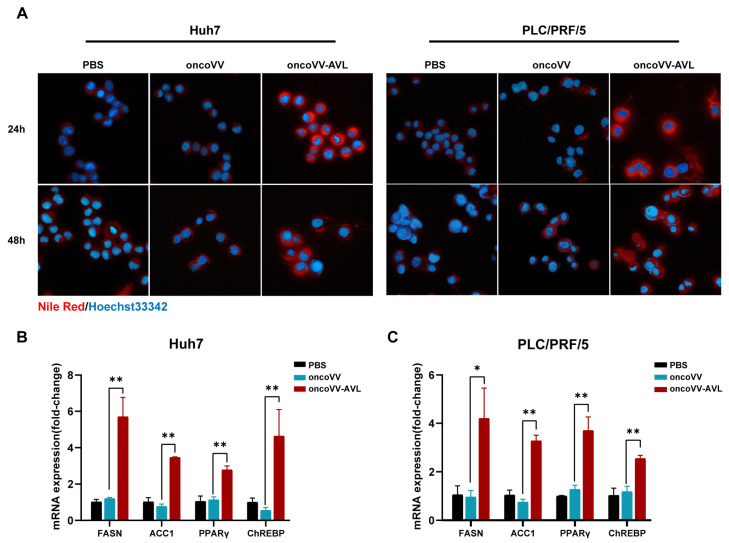
OncoVV-AVL induces lipid accumulation in HCC cells. (**A**) Nile Red staining of lipid droplets (red) in Huh7 and PLC/PRF/5 cells post oncoVV-AVL infection; (**B**,**C**) qPCR analysis of lipogenic genes (FASN, ACC1, PPARγ, and ChREBP) mRNA in Huh7 (**B**) and PLC/PRF/5 (**C**). Data are presented as mean ± SEM; * *p* < 0.05, ** *p* < 0.01.

Initially, we detected the cytotoxicity and virus titer of oncoVV-AVL in HCC cells. Consistent with our previous findings [7], oncoVV-AVL exhibited a potent cytotoxicity against Huh7 and PLC/PRF/5 cells (Figure S1). Notably, Nile Red staining revealed increased lipid accumulation in oncoVV-AVL-infected vs. control cells both at 24 h and 48 h post infection (Figure 1A). Correspondingly, lipogenic genes, including FASN, ACC1, PPAR, and ChREBP1, showed a 2- to 7-fold mRNA upregulation in oncoVV-AVL-infected cells (Figure 1B,C, *p* < 0.01). TCID50 assays demonstrated progressive viral replication at 24 h, 36 h, and 48 h post oncoVV-AVL infection (Figure S2).

### 2.2. OncoVV-AVL Triggers Autophagy in HCC Cells

**Figure 2:**
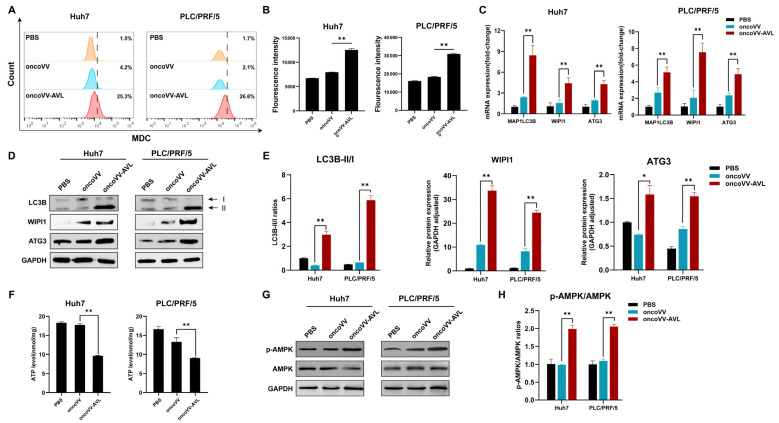
OncoVV-AVL activates AMPK-dependent autophagy in HCC. (**A**,**B**) Flow cytometer quantification of autophagosomes via MDC staining in Huh7 and PLC/PRF/5 cells; (**C**) qPCR analysis of autophagy genes (MAP1LC3B, WIPI1, and ATG3) mRNA in Huh7 and PLC/PRF/5; (**D**,**E**) the expressions of LC3B, WIPI1, and ATG3 were determined by Western blot, band intensities were quantified using Image-J 1.51 software; (**F**) ATP levels in Huh7 and PLC/PRF/5; and (**G**,**H**) the phosphorylation of AMPK quantified by analyzing it in HCC cells. Data are presented as mean ± SEM, * *p* < 0.05, ** *p* < 0.01.

MDC staining and flow cytometry revealed 6–12-fold increases in autophagic cells following oncoVV-AVL treatment (Figure 2A,B). Subsequently, autophagy-related genes were investigated. The qPCR assay revealed the upregulation of LC3B-II (5.1- to 8.4-fold), WIPI (4.4- to 7.6-fold), and ATG3 (4.3- to 4.9-fold) in oncoVV-AVL-infected cells (Figure 2C), and Western blotting confirmed this result (Figure 2D,E). Autophagy is typically activated in response to metabolic stress or other environmental pressures. As an energy sensor, AMP-activated protein kinase (AMPK) plays pivotal roles in growth regulation and metabolic reprogramming, becoming activated when intracellular ATP levels drop. Given the potential induction of metabolic stress by oncoVV-AVL infection and the role of AMPK/ATP in autophagy regulation, we measured the intracellular ATP levels and AMPK activation status (including phosphorylated AMPK) in infected cells. Following oncoVV-AVL infection, ATP levels decreased by 32–45% (Figure 2F), accompanied by obviously elevated p-AMPK/AMPK ratios (Figure 2G,H). These findings collectively suggest that oncoVV-AVL infection induces energy stress, leading to AMPK activation and subsequent autophagy induction.

### 2.3. OncoVV-AVL Results in Glucose Deficiency by Inhibiting GLUTs

**Figure 3:**
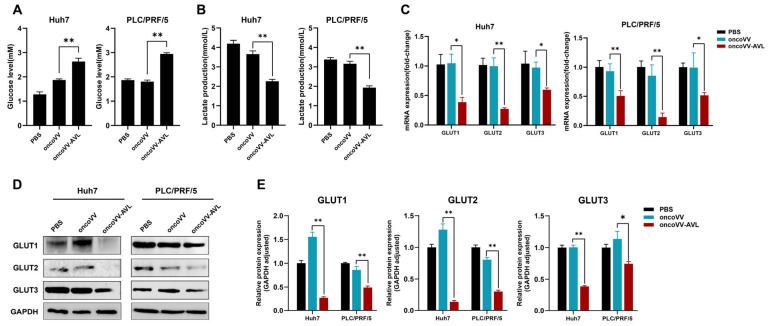
OncoVV-AVL disrupts glucose metabolism in HCC. (**A**) Extracellular glucose levels in Huh7 and PLC/PRF/5; (**B**) lactate production in Huh7 and PLC/PRF/5; (**C**) qPCR analysis of glucose transporters (GLUT1/2/3) mRNA in Huh7 and PLC/PRF/5; and (**D**,**E**) Western blot of GLUT1/2/3 and its quantified analysis. Data are presented as mean ± SEM, * *p* < 0.05, ** *p* < 0.01.

The observed enhancement in lipogenesis, coupled with a reduction in ATP content, prompted us to further investigate glucose metabolism in oncoVV-AVL-infected cells. The results showed that oncoVV-AVL-infected cells exhibited 41–63% higher extracellular glucose (Figure 3A) but 33–34% lower lactate production (Figure 3B), indicating impaired cellular glucose uptake. qPCR and Western blot showed significant reductions in GLUT1, GLUT2, and GLUT3 (GLUT1/2/3) expression (Figure 3C–E). This glucose deprivation phenotype induced by oncoVV-AVL correlated with observed metabolic shifts.

### 2.4. ACSS2 Mediates the Regulation of Autophagic Flux and Lipogenesis in Oncolytic Virotherapy

**Figure 4:**
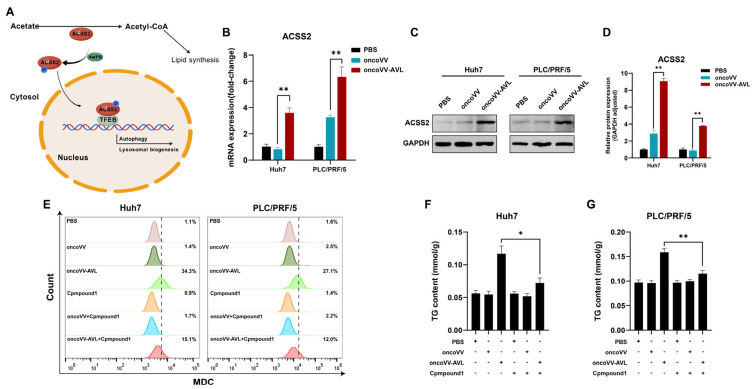
ACSS2 mediates autophagy–lipogenesis crosstalk in oncoVV-AVL treatment. (**A**) Schematic of ACSS2’s dual role; (**B**) evaluation of ACSS2 at mRNA level; (**C**,**D**) the expressions of ACSS2 at protein level and its quantified analysis; (**E**) autophagy inhibition by ACSS2 inhibitor Compound **1** (MDC staining); and (**F**,**G**) TG contents of Huh7 (**F**) and PLC/PRF/5 (**G**). Data are presented as mean ± SEM; * *p* < 0.05, ** *p* < 0.01.

Under glucose deprivation, ACSS2 is phosphorylated by AMPK. This phosphorylation promotes ACSS2’s nuclear translocation and subsequent binding to TFEB, thereby enhancing lysosomal biogenesis and autophagy. Given ACSS2′s established role in mediating the response to glucose deprivation (Figure 4A) [14], we investigated its potential involvement in the effects mediated by oncoVV-AVL. We first compared its expression across treatment groups. Notably, oncoVV-AVL markedly upregulated ACSS2’s expression at both the transcriptional (Figure 4B) and translational levels (Figure 4C,D). Conversely, the pharmacological inhibition of ACSS2 using Compound **1** (10 μM) substantially abrogated oncoVV-AVL-induced autophagy (Figure 4E) and concurrently attenuated lipid accumulation in HCC cells (Figure 4F,G). These data collectively demonstrate that ACSS2 mediates the dual regulation of autophagic flux and lipogenesis during oncoVV-AVL treatment.

### 2.5. OncoVV-AVL Induces the Formation of ACSS2/TFEB Complex

**Figure 5:**
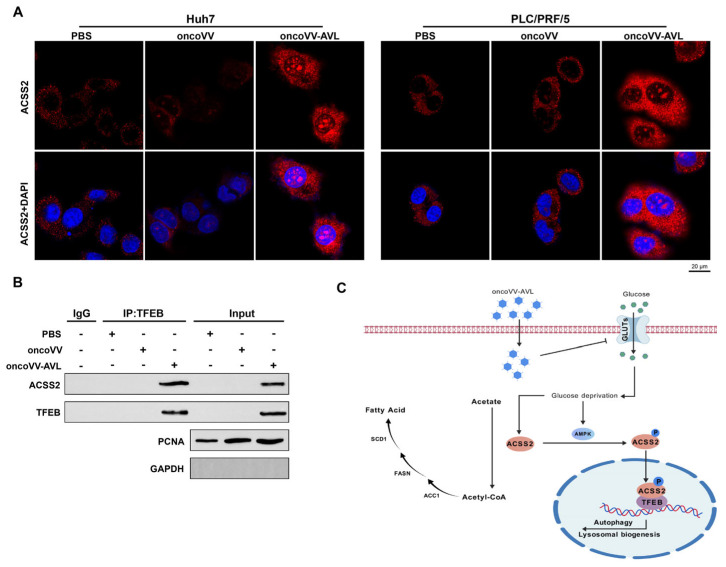
OncoVV-AVL drives ACSS2/TFEB complex formation. (**A**) Confocal microscopy of ACSS2 nuclear translocation (red: ACSS2; blue: DAPI; scale bar: 20 μm); (**B**) co-IP assay showing enhanced ACSS2/TFEB interaction; and (**C**) proposed model of ACSS2/TFEB axis coupling lipogenesis and autophagy.

To delineate the spatiotemporal dynamics of this metabolic–transcriptional axis, we tracked ACSS2 subcellular localization post infection. Strikingly, confocal microscopy revealed the robust nuclear translocation of ACSS2 upon oncoVV-AVL infection (Figure 5A). This spatial redistribution was functionally coupled with strengthened ACSS2/TFEB interaction, as evidenced by co-immunoprecipitation assays showing an increase in binding affinity compared to controls (Figure 5B). These findings prompted us to conclude that oncoVV-AVL inhibits glucose uptake via GLUT1/2/3 downregulation, leading to an energy crisis (ATP depletion) and AMPK activation. This metabolic stress drives ACSS2 nuclear translocation and TFEB binding, which coordinately upregulate autophagy genes (LC3B, WIPI, and ATG3) and lipogenic factors (FASN, ACC1, and SCD1). Thus, oncoVV-AVL reprograms HCC metabolism by inducing ACSS2/TFEB-driven autophagy and lipid metabolic switching, which synergize with its direct oncolytic activity (Figure 5C).

### 2.6. Metabolic Modulation via ACSS2 Enhances the Antitumor Efficacy of OncoVV-AVL in HCC Xenograft Models

**Figure 6:**
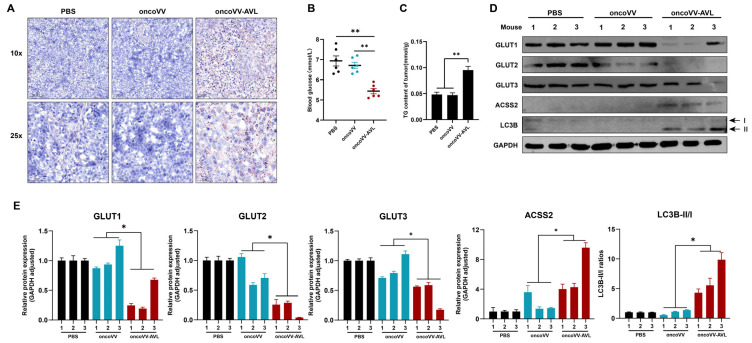
ACSS2-mediated metabolic reprogramming enhances antitumor efficacy of oncoVV-AVL in xenografts. (**A**) Oil Red O staining of tumor sections; (**B**) serum glucose level of mice; (**C**) tumor TG content; and (**D**,**E**) the expressions of GLUT1/2/3, ACSS2, and LC3B in tumor tissues, band intensities were quantified by Image-J, Western blotting analysis was performed on individual tumor lysates from each mouse (n = 3 per group). Data are presented as mean ± SEM; * *p* < 0.05, ** *p* < 0.01.

To validate the relevance of ACSS2-mediated metabolic modulation, we performed analyses in Huh7-derived xenografts. The mice were intratumorally injected with oncoVV-AVL or oncoVV, with PBS as the negative control. Consistent with our previous study, mice receiving intratumoral oncoVV-AVL exhibited marked tumor growth suppression compared to the PBS and oncoVV controls (Figure S3), paralleled by profound metabolic remodeling. The oncoVV-AVL-treated tumors displayed increased lipid droplets, which were stained by Oil Red O (Figure 6A), and a 2-fold elevated TG content (Figure 6C). Meanwhile, the serum assessment showed a 42% reduction in circulating glucose (*p* < 0.01) (Figure 6B), as well as concomitant ACSS2 upregulation and GLUT1/2/3 downregulation (Figure 6D,E). Elevated LC3B-II/I ratios confirmed autophagy activation induced by oncoVV-AVL administration in the xenograft models (Figure 6D,E). These in vivo results collectively establish ACSS2 as a central node coordinating lipid anabolism and autophagic flux during oncoVV-AVL-mediated antitumor responses.

## 3. Discussion

As versatile anticancer agents with a growing efficacy, oncolytic viruses (OVs) [16] function through the following four primary mechanisms: oncolysis, vascular targeting, transgene expression, and virus-induced antitumor immune responses [16,17,18]. Our study elucidates the novel mechanism by which oncoVV-AVL induces metabolic reprogramming in HCC through the ACSS2/TFEB axis, bridging viral oncolysis with host lipid–autophagy crosstalk. Our findings expand the mechanistic repertoire of OVs to include metabolic modulation, a strategy akin to pathogenic viruses like HBV and SARS-CoV-2, which hijack host lipid metabolism for replication [9,10,11]. However, unlike these pathogens, oncoVV-AVL exploits this metabolic vulnerability to destabilize tumor survival networks.

The ability to overcome metabolic stress is crucial for tumor development. In response, cancer cells develop extra adaptive mechanisms for metabolic stress survival, for instance, incorporating acetate as a secondary carbon substrate [19]. Acetyl-CoA generated from glucose and acetate uptake is important for histone acetylation and gene expression. Studies have shown that ACSS2, which is often upregulated in some tumors, participates in the metabolic rewiring of tumor cells in order to maintain acetyl-CoA levels [20]. Under glucose limitation, acetate activates ACSS2 to mediate SNA1 histone acetylation, a key EMT (epithelial to mesenchymal transition) driver, thereby promoting renal cell carcinoma metastasis [21]. However, how acetyl-CoA is produced post OV infection is unclear.

We demonstrate here that glucose deprivation results in the AMPK-mediated nuclear translocation of ACSS2. Key to this process is ACSS2, which we identified as a dual regulator of lipogenesis and autophagy. Under glucose deprivation induced by oncoVV-AVL (via GLUT1/2/3 suppression), AMPK activation triggered ACSS2 nuclear translocation, where it partnered with TFEB to synchronize lipid synthesis (FASN/ACC1 upregulation) and autophagic flux (LC3B-II/I elevation). This paradoxical coupling—simultaneously fueling viral replication (via lipid provisioning) and destabilizing tumor metabolism—represents an evolutionary advantage for OVs. Our data align with reports linking ACSS2 to stress-induced autophagy [14], yet uniquely position it as a rheostat for OV-mediated metabolic warfare.

Although this study establishes oncoVV-AVL as a potent metabolic modulator, we recognize that the isolated contributions of AVL warrant deeper investigation. The critical role of AVL was evidenced by the failure of the oncoVV control (lacking AVL) to inhibit GLUT expression. Studies report that galectin-1 regulates angiogenesis-related genes via RNA binding [22], while its complex formation with HOXA5 reprograms transcriptional networks in brain tumor stem cells [23]. Although these studies establish that carbohydrate-binding proteins modulate transcriptional landscapes, our findings highlight a distinct mechanism mediated by the heterologous C-type lectin AVL. Notably, AVL differs significantly from endogenous galectins in both structure and glycan-binding specificity. Native AVL cannot penetrate tumor cells efficiently due to membrane impermeability. Therefore, our study design prioritized oncoVV-AVL as an integrated therapeutic platform where oncolytic virus serves as tumor-targeted delivery vehicle. The precise mechanism through which AVL’s carbohydrate recognition domain interfaces with GLUT suppression remains to be elucidated and represents a key focus for future investigation into its antitumor activity. Future studies employing advanced AVL delivery modalities will disentangle its direct transcriptional effects from viral context-dependent actions.

While our current study establishes the functional link between AVL and GLUT suppression, the precise molecular mechanism by which AVL’s carbohydrate-binding capability interfaces with this suppression remains an active area of investigation. Future research will specifically address this mechanism.

The translational implications are twofold. First, the observed in vivo metabolic remodeling—marked by hypoglycemia, hypoinsulinemia, and lipid-rich tumors—suggests systemic metabolic stress as a biomarker for OV efficacy. Second, the vulnerability of HCC cells to ACSS2 inhibition (Compound **1**) highlights a therapeutic window for combining oncoVV-AVL with ACSS2-targeted agents. Notably, our xenograft data mirror clinical observations in HBV-associated HCC, where lipogenic enzyme inhibitors synergize with antivirals [2], supporting the translational relevance of this approach.

There are still some limitations in this study. Our study focused on Huh7 and PLC/PRF/5 cell models, but HCC heterogeneity warrants validation in patient-derived organoids. Additionally, the role of TFEB in immune modulation (e.g., cytokine secretion) during virotherapy remains unexplored. Future work should investigate AMPK agonists (e.g., metformin) to amplify oncoVV-AVL’s metabolic effects and assess toxicity in immunocompetent models.

## 4. Materials and Methods

### 4.1. Cell Culture and Viral Engineering

Human HCC cell lines (Huh7 and PLC/PRF/5) and HEK 293A (Meisen, Hangzhou, China) were maintained in DMEM medium (Gibco, Carlsbad, CA, USA) with 10% FBS (Hyclone, South Logan, UT, USA), 4 mM L-glutamine, and 50 U/mL penicillin–streptomycin at 37 °C under 5% CO_2_. Mycoplasma contamination was routinely excluded via PCR. The thymidine kinase (TK)-deleted vaccinia virus WR strain (ATCC) was used to construct oncoVV through homologous recombination. The recombinant oncoVV-AVL, expressing Aphrocallistes vastus lectin (AVL), was generated as previously reported [24]. Viral stocks were propagated in HEK 293A cells and purified by three freeze–thaw cycles with centrifugation (1000× *g*, 10 min).

### 4.2. Viral Replication Assay

Viral titers were determined by TCID50 assay, as previously described [7]. Briefly, cells in 96-well plates were infected with serial 10-fold viral dilutions. Supernatants harvested at 12, 24, and 48 h post infection were titrated on HEK 293A monolayers.

### 4.3. Subcellular Localization of ACSS2

Lipid droplets were stained with 0.75 μg/mL Nile Red (Sigma-Aldrich, St. Louis, MO, USA) for 15 min at RT and imaged using an Olympus IX73 microscope (Ex/Em: 485/535 nm). Autophagic flux was assessed by incubating cells with 50 μM monodansylcadaverine (MDC, Beyotime) for 30 min at 37 °C, followed by flow cytometry (BD Accuri C6; Ex/Em: 330/525 nm).

### 4.4. Lipid Formation and Autophagy Analysis

To monitor the location of ACSS2, cells were seeded on glass-bottom dishes, fixed with 4% paraformaldehyde at 36 h post viral infection, permeabilized with 0.1% Triton X-100, and immunostained with Alexa Fluor 488-conjugated anti-ACSS2 (1:200, Abcam ab133664, Cambridge, UK). Nuclei were counterstained with DAPI. Images were acquired using a confocal microscope (Olympus FV3000, Tokyo, Japan).

### 4.5. Biochemical Examination

Intracellular triglycerides (TGs) and total cholesterol (TC) were quantified using commercial kits (Nanjing Jiancheng, Nanjing, China) after cell lysis with 2% Triton X-100. For xenograft analysis, 100 mg tumor tissues were homogenized in ethanol (1:9 *w*/*v*), and the supernatant was analyzed per TG/TC kit protocols. Serum insulin levels from orbital blood samples were measured by ELISA (Beyotime, Shanghai, China). Glucose consumption and lactate production were determined using assay kits (Beyotime/Solarbio). ATP levels were quantified with a bioluminescence kit (Beyotime).

### 4.6. Xenograft Tumor in Nude Mice

All animal procedures were approved by the Institute Animal Care and Use Committee (IACUC) of Zhejiang Sci-Tech University (Protocol number:20230313102). Female Balb/c nude mice (6–7 weeks, Slack Laboratory, Shanghai, China) were accommodated under standardized conditions (18–23 °C, 50% humidity). The mice were inoculated subcutaneously with 5 × 10^6^ Huh7 cells, and then were randomized into PBS, oncoVV (1 × 10^7^ PFU), and oncoVV-AVL (1 × 10^7^ PFU) groups (n = 6) when tumors reached 200 mm^3^. Tumor volumes (length × diameter^2^ × 0.5) were monitored every 2 days. Terminal tumors were processed for histology or snap-frozen.

### 4.7. RNA Isolation and RT-qPCR Analysis

Total RNA extracted with TRIzol (Sigma-Aldrich, St. Louis, MO, USA) was reverse-transcribed using Ready-to-go beads (GE Healthcare, Chicago, IL, USA). RT-qPCR was performed with SYBR Green Master Mix (Toyobo, Osaka, Japan) using the primers listed in Table S1, with GAPDH normalization and 2^-ΔΔCt analysis.

### 4.8. Immunologic Blots Analysis

For immunoblotting, proteins separated by 12% SDS-PAGE were transferred to PVDF membranes (Millipore, MA, USA), blocked with 5% skim milk, and probed with primary antibodies (4 °C overnight) followed by HRP-conjugated secondaries. Signals were detected using ECL (PerkinElmer, Waltham, MA, USA) and visualized on a Clinx 6000EXP system. Antibodies are listed in Table S2. Co-immunoprecipitation (Co-IP) assay used anti-TFEB (Abcam, Cambridge, UK) with protein A/G agarose beads (Beyotime), with IgG serving as a negative control. P Complexes were analyzed by Western blot.

### 4.9. Histological Staining

Frozen tumors were embedded in OCT and sliced into 6–8 μm sections, which were further stained with freshly prepared Oil Red O (0.3% in 60% isopropanol) for 60 min at 37 °C and counterstained with hematoxylin for 2 min. The images were acquired at 40× magnification (Nikon Eclipse Ti, Tokyo, Japan).

### 4.10. Statistical Analysis

Data are presented as mean ± SEM. The comparisons were performed with the Mann–Whitney U test between two groups. One-way ANOVA with Tukey’s post hoc test was used for comparing three or more groups. *p* < 0.05 was considered as statistically significant.

## Data Availability

Data are contained within the article.

## Supplementary Materials

The following supporting information can be downloaded at: https://www.mdpi.com/article/10.3390/md23080297/s1, Table S1. List of primers used in this study; Table S2. List of antibodies used in this study; Figure S1. The cytotoxic effect of oncoVV-AVL on pancreatic cancer cells; Figure S2. OncoVV-AVL promotes viral reproduction; Figure S3. Tumor volume curve.
