# Supplementary material for: An Oncolytic Vaccinia Virus Expressing Aphrocallistes Vastus Lectin Modulates Hepatocellular Carcinoma Metabolism via ACSS2/TFEB-Mediated Autophagy and Lipid Accumulation
Source: Mar Drugs. 2025 Jul 24;23(8):297. doi: 10.3390/md23080297 (PMC12387248; doi:10.3390/md23080297)
Supplement: Supplementary file 1 [file marinedrugs-23-00297-s001.zip › marinedrugs-3711522-supplementary.pdf]

Supplementary information for

An oncolytic vaccinia virus expressing Aphrocallistes vastus lectin modulates hepatocellular carcinoma metabolism via ACSS2/TFEB-mediated autophagy and lipid accumulation

Supplementary Tables

Supplementary Table S1. List of primers used in this study

Primers	Sequence(Forward, 5′-3′)	Sequence(Reverse, 5′-3′)
**ACSS2**	GGATCACTGGTCATTCCTAC	GTGCTGTGTAGAACTTGGTC
**MAP1LC3B**	GTCTATGCCTCCCAGGAGAC	GATGAACTGATCGATCTCAGTTGG
**ATG3**	CAGATGAATTGGAAGCTATC	CATAGTGCTGAGCAATCTTG
**WIPI**	CATGAGTTTGCGACGGGACCAG	GATGTCCTGATTTCTGAGGTGTGC
**SLC2A1/Glut1**	CCTGCAGTTTGGCTACAACA	GTGGACCCATGTCTGGTTG
**SLC2A2/Glut2**	AGCTTTGCAGTTGGTGGAAT	CCCATCAAGAGAGCTCCAAC
**SLC2A3/Glut3**	TCATTTCCATTGTGCTCCAG	GGCATAGATGGGCTCTTGAA
**GAPDH**	GACAGTCAGCCGCATCTTCT	GCGCCCAATACGACCAAATC

Supplementary Table S2. List of antibodies used in this study

Antibody	Article Number	Dilution ratio
Anti-GAPDH	cst.5174S	1:5000
Anti-WIPI-1	cst.12124S	1:2000
Anti-ATG3	A5809	1:2000
Anti-LC3B	cst.2775S	1:2000
Anti-AMPK	cst.2532S	1:2000
Anti-Phospho-AMPK	cst.50081S	1:2000
Anti-GLUT1/SLC2A1	A6982	1:2000
Anti-GLUT2/SLC2A2	A12307	1:2000
Anti-GLUT3/SLC2A3	A4137	1:2000
Anti-ACSS2	ab133664	1:2000 (1:200)
Anti-TFEB	ab267351	1:2000
Goat Anti-Rabbit IgG(H+L)	AS014	1:10000
Goat Anti-Mouse IgG(H+L)	AS003	1:10000

Supplementary Figures

Supplementary Fig.S1

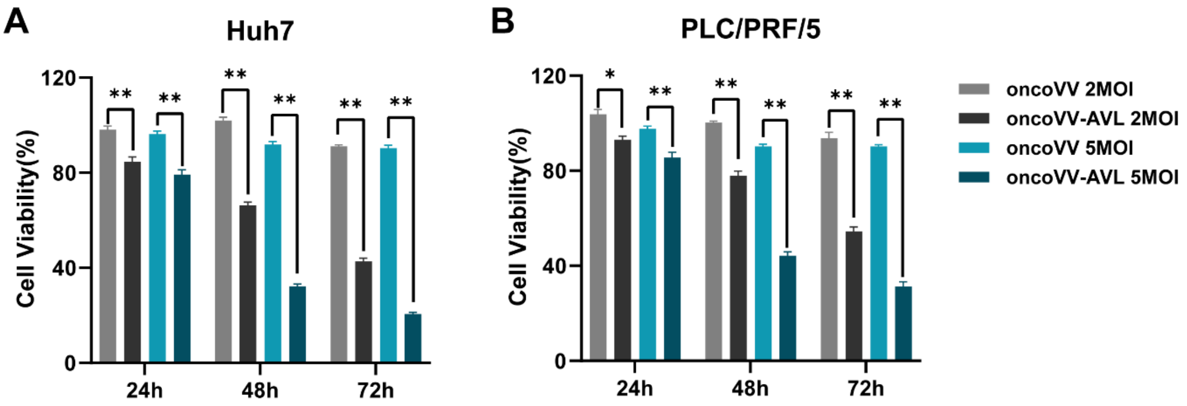

Fig.S1. The cytotoxic effect of oncoVV-AVL on pancreatic cancer cells. Cell viability was assessed in Huh7 and PLC/PRF/5 cells after infection with oncoVV or oncoVV-AVL at various MOIs of 2, and 5 at 24 h, 48 h, and 72 h.

Supplementary Fig.S2

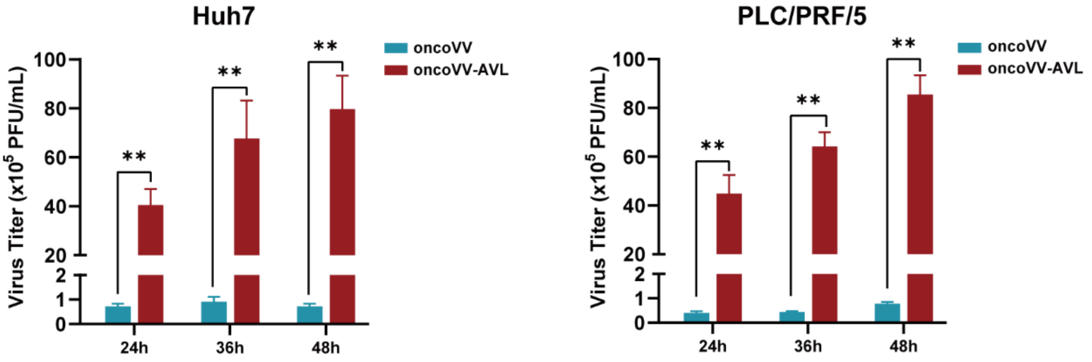

Fig.S2. OncoVV-AVL promotes viral reproduction. Viral yields in Huh7 and PLC/PRF/5 cells.

Supplementary Fig.S3

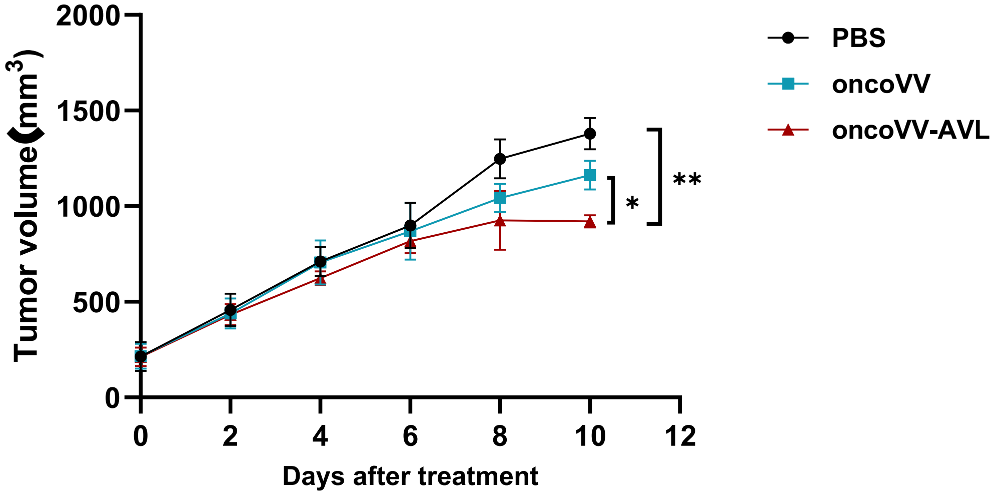

Fig.S3. Tumor volume curve. The measurement of tumor volume occurred at 2-day intervals. Data are expressed as mean tumor volume ± SEM (n = 6, *P < 0.05, **P < 0.01).
